# Infrapatellar Fat Pad-Synovial Membrane Anatomo-Fuctional Unit: Microscopic Basis for Piezo1/2 Mechanosensors Involvement in Osteoarthritis Pain

**DOI:** 10.3389/fcell.2022.886604

**Published:** 2022-06-28

**Authors:** Aron Emmi, Elena Stocco, Rafael Boscolo-Berto, Martina Contran, Elisa Belluzzi, Marta Favero, Roberta Ramonda, Andrea Porzionato, Pietro Ruggieri, Raffaele De Caro, Veronica Macchi

**Affiliations:** ^1^ Department of Neuroscience, Section of Human Anatomy, University of Padova, Padova, Italy; ^2^ Musculoskeletal Pathology and Oncology Laboratory, Department of Surgery, Oncology and Gastroenterology (DiSCOG), University of Padova, Padova, Italy; ^3^ Orthopedics and Orthopedic Oncology, Department of Surgery, Oncology and Gastroenterology (DiSCOG), University of Padova, Padova, Italy; ^4^ Rheumatology Unit, Department of Medicine-DIMED, University of Padova, Padova, Italy; ^5^ Internal Medicine I, Cà Foncello Hospital, Treviso, Italy

**Keywords:** Piezo1/2, anatomo-functional unit, infrapatellar fat pad, synovial membrane, knee, osteoarthritis, pain, biomechanics

## Abstract

The Infrapatellar Fat Pad (IFP) is a fibro-adipose tissue of the knee recently reconsidered as part of a single anatomo-functional unit (AFU) together with the synovial membrane (SM). Several evidence support the role of this unit in the mechanisms that trigger and perpetuate the onset and progression of osteoarthritis (OA) disease. Additionally, the contribution of IFP-SM AFU in OA-associated pain has also been supposed, but this assumption still needs to be fully elucidated. Within this context, the recent discovery of the mechanoceptive Piezo ion channels (i.e., Piezo1 and Piezo2) in mammals and consciousness on their role in mediating both mechanoceptive and inflammatory stimuli could shed some light on knee OA pain, as well as on the process leading from acute to chronic nociceptive responses. For this purpose, the IFP-SM AFUs of both healthy donors (non-OA IFP-SM AFUs, *n* = 10) and OA patients (OA IFP-SM AFUs, *n* = 10) were processed by histology and immunohistochemistry. After the attribution of a histopathological score to IFP-SM AFUs to confirm intrinsic differences between the two groups, the specimens were investigated for the expression and localization/distribution pattern of the mechanosensors Piezo1 and Piezo2. In addition, the presence of monocytes/macrophages (CD68), peripheral nerve endings (PGP9.5) and neoangiogenesis signs (YAP1) was evaluated for a broad tissue characterization. The study results lead to a better description of the IFP-SM AFU microscopic features in both healthy and pathological conditions, highlighting peculiar differences in the study cohort. Specifically, immunopositivity towards Piezo1/2, CD68 and YAP1 markers was detected at vessels level in the OA- IFP-SM AFUs compartments, differently from the non-OA-group. A correlation with pain was also inferred, paving the way for the identification of new and effective molecules in OA management.

## 1 Introduction

Knee osteoarthritis (OA) is a multifactorial and progressive whole joint disease ultimately leading to cartilage/bone loss (Loeser et al., 2012; Belluzzi et al., 2017; Belluzzi et al., 2019b; Stocco et al., 2022b). The defining clinical presentation is pain, associated with stiffness, loss of motion and subsequent functional disability (Syx et al., 2018; Raeissadat et al., 2020; Zhang et al., 2020). Specifically, two different types of OA pain can be identified: an intermittent but severe/intense pain and a persistent background pain or aching (Neogi, 2013). Despite the possibility to recur to different FDA-approved treatment options, ranging from anti-inflammatory drugs (NSAIDs), viscosupplementation or steroids injections (Favero et al., 2021) and, even to antidepressants as pain threshold modulators (Wang et al., 2015; Ferreira et al., 2021), the control over OA symptoms with pain relief remains largely inadequate, representing the major reason for pursuing joint replacement surgery (Syx et al., 2018; Stocco et al., 2022a).

To date, OA pathology and progression factors have been examined in detail, but the “actors” and mechanisms involved in OA pain remain poorly understood (Ter Heegde et al., 2019) resulting in scarce efficiency in identifying adequate strategies for its management (Lane et al., 2011; Belluzzi et al., 2019b). Mechanical loading, including compression, tension, shear, and implying physicochemical changes (i.e., fluctuations in osmolarity) has a critical role over the physiopathological features of healthy or diseased synovial joints, being responsible for the transformation of external biophysical stimuli into intracellular biochemical signals, in turn determining or affecting cells behaviour (Lee et al., 2017; Dieterle et al., 2021). While the effects of mechanostimulation and mechanotransduction have been long investigated in cartilage tissue, there is a lack of information regarding the other joint tissues that are actively involved in OA (Abramson and Attur, 2009; Martel-Pelletier and Pelletier, 2010; Dieppe, 2011). In recent years many efforts were directed towards a full comprehension of the knee anatomy with increasing consciousness that OA understanding cannot be separated from anatomy itself (Macchi et al., 2016). Within this scenario, a new emerging idea reconsidering the relation between the infrapatellar fat pad (IFP) and the adjacent synovial membrane (SM) aroused. In fact, several evidences based on macroscopic anatomy, imaging, histopathology (the IFP and SM) show increase of inflammatory features in OA (Klein-Wieringa et al., 2016) and molecular biology, highlight the tight interaction among these structures, supporting the existence of a IFP-SM anatomo-functional unit (AFU) (Macchi et al., 2018). Despite physiological age-related modifications (Stocco et al., 2021) the AFU seems to be actively involved in OA pathogenesis and pain (Macchi et al., 2018; Belluzzi et al., 2019a).

Piezo receptors (Piezo1 and Piezo2) are cation-permeable ion channels that sense mechanical stimuli (such as blood flow, light touch, and proprioception) and may transduce information about injurious mechanical stress to chondrocytes in articular cartilage. Particularly, they mediate high-strain hyperphysiologic loading that is a high risk factor of OA (Lee et al., 2017). Considering these mechanoreceptors, it may be possible that, similarly to cartilage tissue (Lee et al., 2014; Lee et al., 2021), also the IFP-SM AFU becomes more sensitive to mechanical stimulation through their involvement (whether present), thus acting as peculiar “pain probes” in OA. Additionally, investigation on their eventual combined expression may also elucidate their synergic effect on the specific physiological response they mediate (Lee et al., 2014). Therefore, for the first time to our knowledge, the presence and distribution pattern of Piezo receptors in the IFP-SM AFU of healthy and OA subjects was here considered by microscopic and immunohistochemical analyses. Moreover, presence and distribution of specific markers for monocyte/macrophages, implying the existence of an inflammatory condition; nerve endings, suggesting neuropathic pain; vessels endothelial cells, associated to angiogenesis and tissue remodeling, were also assessed in the whole cohort to broadly describe the typical anatomical and physiopathological features associated to the IFP-SM AFU in case of OA-disease.

To date there is the urgent and pressing need to unravel not only the etiopathogenesis but also the pathways leading to OA pain (Belluzzi et al., 2019b). Clarifying presence and distribution of Piezo1/2 receptors within the IFP-SM AFU pathological environment may guide towards the identification of specific targets for the development of new effective treatments for OA pain management. Moreover, these results will possibly lay the basis for future functional studies *in vitro*, better describing Piezo 1/2 receptors’ role *in vivo* as well as eventual correlations with cartilage specific features in OA.

## 2 Materials and Methods

### 2.1 Study Groups Characteristics

Two different groups were considered in this study: the non-OA IFP-SM AFU and OA- IFP-SM AFU groups.

For the non-OA IFP-SM AFUs group, samples (*n* = 10) were collected from not-embalmed cadavers enrolled in the Body Donation Program “Donation to Science” of the University of Padova (Porzionato et al., 2012; Porzionato et al., 2018; Boscolo-Berto et al., 2020). In accordance with the inclusion/exclusion criteria, no history of symptomatic OA, knee comorbidities, rheumatic or inflammatory disorders, tumors, signs of cartilage degradation or presence of osteophytes on dissection was reported.

As for sampling, full thickness longitudinal specimens (from the patellar tendon to the bone aspect) were excised from the apex of the patella up to the tibial tuberosity, without dissecting the SM which was preserved. Hence, the samples were fixed in 10% formalin solution, paraffin embedded according to routine protocols and processed for the histological/immunohistochemical analyses described below. Regarding the IFP-SM OA-AFUs group, samples (*n* = 10) were excised from patients undergoing elective total knee arthroplasty (TKA) (Kellgren-Lawrance 3-4) at the Orthopedics and Orthopedic Oncology, University-Hospital of Padova (Macchi et al., 2011; Belluzzi et al., 2019a). Good health, without tumor and/or knee comorbidities as well as absence of infective or inflammatory conditions constituted the prerequisites of eligibility. This study was performed after receiving institutional review board approval (CESC Code: 4510/AO/18); the study protocol did not include cartilage tissue sampling. All patients signed written informed consent.

### 2.2 Histopathological Grading of the Infrapatellar Fat Pad-Synovial Membrane Anatomo-Functional Units

To verify and confirm the absence/presence of inflammation, the tissues were preliminarily investigated for detection of eventual inflammatory cells and vessels numerosity. Thus, the histopathological score was applied to IFP and adjacent SM together. Briefly, three consecutive sections/sample of 10 μm in thickness/each were stained with hematoxylin-eosin (H&E). Mononuclear cell infiltration was graded as follows: grade 0 = no presence of mononuclear cell infiltration; grade 1 = presence of perivascular mononuclear cell infiltration; grade 2 = both perivascular and interstitial mononuclear cell infiltration. As for vascularity, the total number of vessels was determined by counting. Three sequential sections/case were considered.

In parallel, on the sections, a morphometric study was also conducted to measure and compare thickness of the SM.

The images were recorded under a Leica DM4500B microscope (Leica Microsystems Wetzlar, Germany) connected to a Leica DFC320 high-resolution digital camera (Leica Microsystems).

### 2.3 Immunohistochemical Characterization of the Infrapatellar Fat Pad-Synovial Membrane Anatomo-Functional Units

The histopathological characterization of the IFP-SM AFUs was then integrated with immunohistochemical analysis. Immunoperoxidase staining was performed on a Dako EnVision Autostainer according to manufacturer recommendations; the immunohistochemical characterization was performed with the following antibodies diluted in PBS: Piezo1 (Polyclonal Rabbit Anti-Human, Invitrogen, Code Number: PA5-106296); Piezo2 (Polyclonal Rabbit Anti-Human, Invitrogen, Code Number: PA5-56894); CD68 (Monoclonal Mouse Anti-Human, Clone L26, Dako Omnis, Code Number: M0756); PGP 9.5 (Monoclonal Mouse Anti-Human, Clone 31A3, Invitrogen, Code Number MA1-83428); Yes-Associated Protein 1 (YAP1) (Polyclonal Rat Anti-Human, Immunological Sciences, Code Number: AB-84335).

All peroxidase reactions were repeated at least three times to ensure reaction consistency.

### 2.4 Morphometric Quantification of the Infrapatellar Fat Pad-Synovial Membrane Anatomo-Functional Units

**FIGURE 1:**
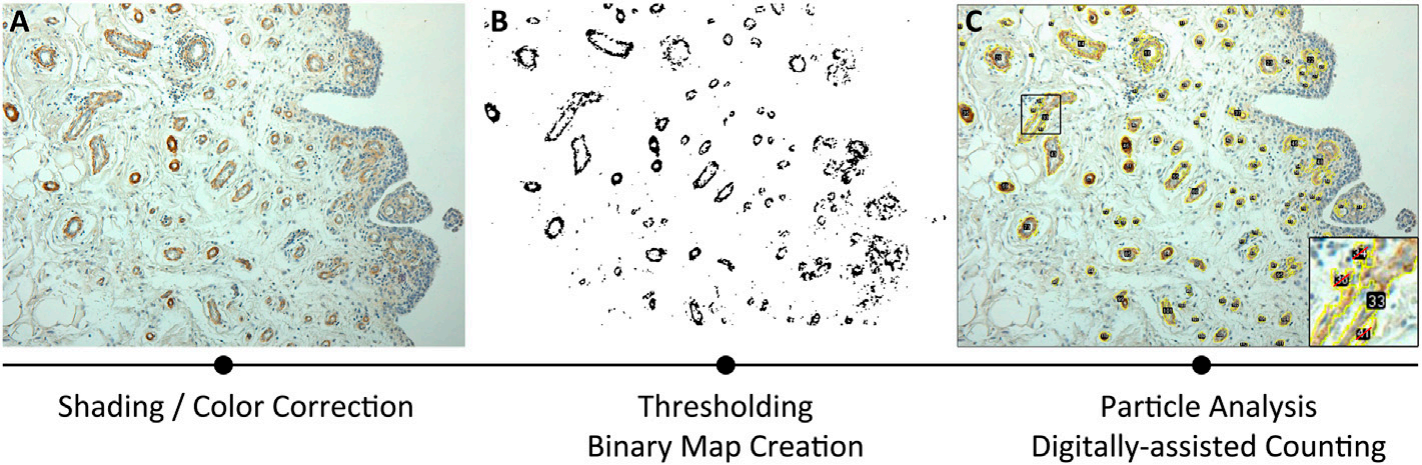
Methodological approach for Piezo receptors morphometrical analysis, based on immunoreactivity. The three consequential steps include: **(A)** shading/color correction of the image; **(B)** thresholding through binary map creation; **(C)** particle analysis by digitally assisted counting.

Photomicrographs were acquired under a Leica DM4500B microscope (Leica Microsystems) connected to a Leica DFC320 high-resolution digital camera (Leica Microsystems). Image processing was performed through softwares for image acquisition (QWin, Leica Microsystems) and analysis (ImageJ), as previously reported (Porzionato et al., 2020; Emmi et al., 2021) (Figure 1).

Methodologically, an average of four ×20 magnification photomicrographs/sample were acquired as counting fields, corrected for shading, and loaded into ImageJ software for semi-automatic immunoreactivity quantification (Figure 1A). Digital photomicrographs were loaded as stack, contrasted and sharpened; hence, a Maximum Entropy Threshold was applied and manually adjusted for each section to discern immunopositive elements from the background and the negative tissue (Figure 1B). A quality control of the applied threshold also occurred by overlying the thresholdered images to the original photomicrographs. Thereafter, particle analysis occurred assigning a 0-infinity px threshold to define quantity of the immunoreactive elements and total area within the digital image, with the range being manually adjusted by the evaluators in accordance to mean particle size; furthermore, to avoid quantification of fragmented single reactivities, manual quality control was performed (Figure 1C). Regarding cellular immunostaining, the mean particle size was estimated and the quantity of the immunoreactive elements was divided by the total area of the sample for a semi-quantitative measure of immunoreactive density within each section. Total immunoreactive area (µm^2^) and the percentage of the immunoreactive area (A%) was also estimated per counting field. Counting fields for each available sample were treated as repeated measures and averaged per single subject.

### 2.5 Statistical Analysis

Statistical analyses were performed by GraphPad Prism 9. Unpaired *t*-test was used to compare continuous variables. Piezo2 immunoreactive vessel density in the synovial and IFP compartments were determined by two-way ANOVA tests with Tukey multiple comparisons test. Differences in CD68^+^ particle size/density between OA and non-OA subjects were analyzed by t tests with Welch’s correction. Pearson’s correlation was performed between the different morphometrical variables in the correlation matrix. Further statistical details for each plot can be found in the corresponding figure legend. Throughout the manuscript * indicates *p* < 0.05, **p < 0.01, ***p < 0.001 and ****p < 0.0001.

## 3 Results

### 3.1 Demographic Data of the Cohort

The mean age of the donors of the non-OA IFP-SM AFU group was 70 years (range, 60–87) (females, *n* = 6; males, *n* = 4), while the mean age of the OA IFP-SM AFU group was 65 years (range, 51–77) (females, *n* = 5; males, *n* = 5) (*p* = 0.52).

The mean body mass index (BMI) was 25.4 kg/m^2^ (range, 22–31) for the non-OA group, whereas 35.41 (range, 29.6–37.9) for the OA-group (*p* = 0.025) (Table 1).

**TABLE 1:** Patient’s demographic data.

	Non-OA AFUs	OA-AFUs
Sample size	*n* = 10	*n* = 10
Sex	Female, *n* = 6 Male, *n* = 4	Female, *n* = 5 Male, *n* = 5
Age, years	70	65
BMI, kg/m^2^ (Mean value)	25.4	35.41

### 3.2 Histopathological Analysis Confirming Inflamed Status in Osteoarthritis-Infrapatellar Fat Pad-Synovial Membrane Anatomo-Functional Units

**FIGURE 2:**
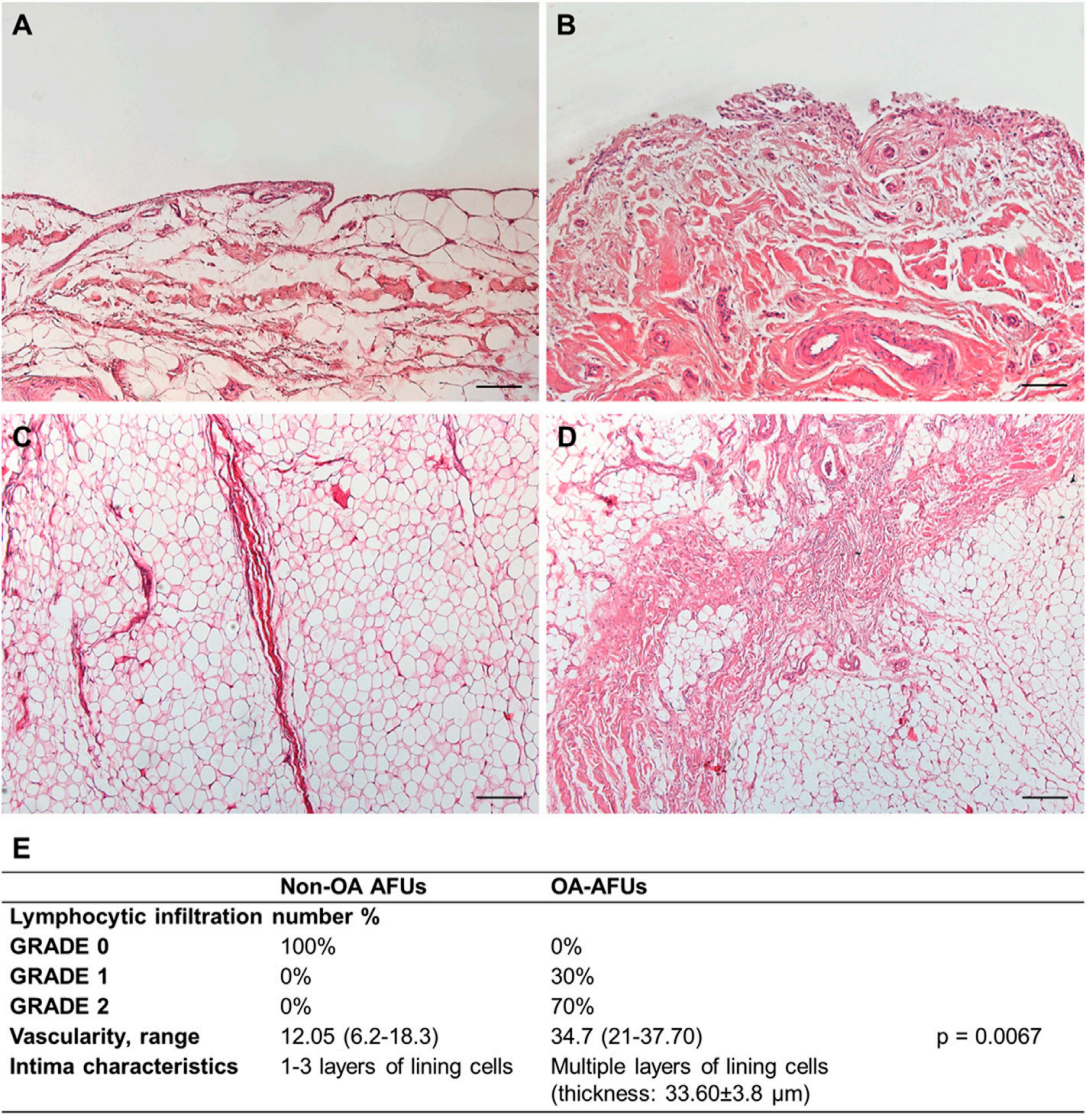
**(A**,**B)** Histological features of the IFP compartments in the non-OA **(A**,**C)** and OA-IFP-SM AFUs **(B**,**D)**; in presence of OA, typical thick septa are identifiable within the adipocytes. **(E)** Table showing the combined IFP-SM AFU histopathological grading results; specifically, lymphocytic infiltration, median vascularity range and intimal layer thickness were considered. Scale bars: 200 µm **(A**,**B)**.

Histopathological evaluation of the IFP-SM AFUs revealed significant differences between the two groups in both inflammatory cells and vessels content (Figures 2A–C).

Mononuclear cell infiltration graded as 0 corresponded to 100% of the non-OA IFP-SM AFUs; whereas, 70 and 30% of the OA-IFP-SM AFUs were graded as 1 and 2, respectively. Considering vessels, the median values/group were 12.8 (range, 6.2–18.3) for the non-OA donors and 34.7 (range, 21–37.70) for the OA-patients (*p* = 0.0067), mainly identifiable in the subintimal layer.

Additionally, while the non-OA SM showed the typical lining layer characterized by one to three layers of lining cells, it appeared as hyperplastic with multiple cell layers in the OA-group. Hence, the increased thickness of the intima was calculated, showing a mean value of 33.60 ± 3.8 µm.

### 3.3 Immunohistochemical Characterization of the Infrapatellar Fat Pad-Synovial Membrane Anatomo-Functional Units Showing Specific Features in Osteoarthritis

#### 3.3.1 Piezo1/2 Mechanoreceptors in the Infrapatellar Fat Pad-Synovial Membrane Anatomo-Functional Units

**FIGURE 3:**
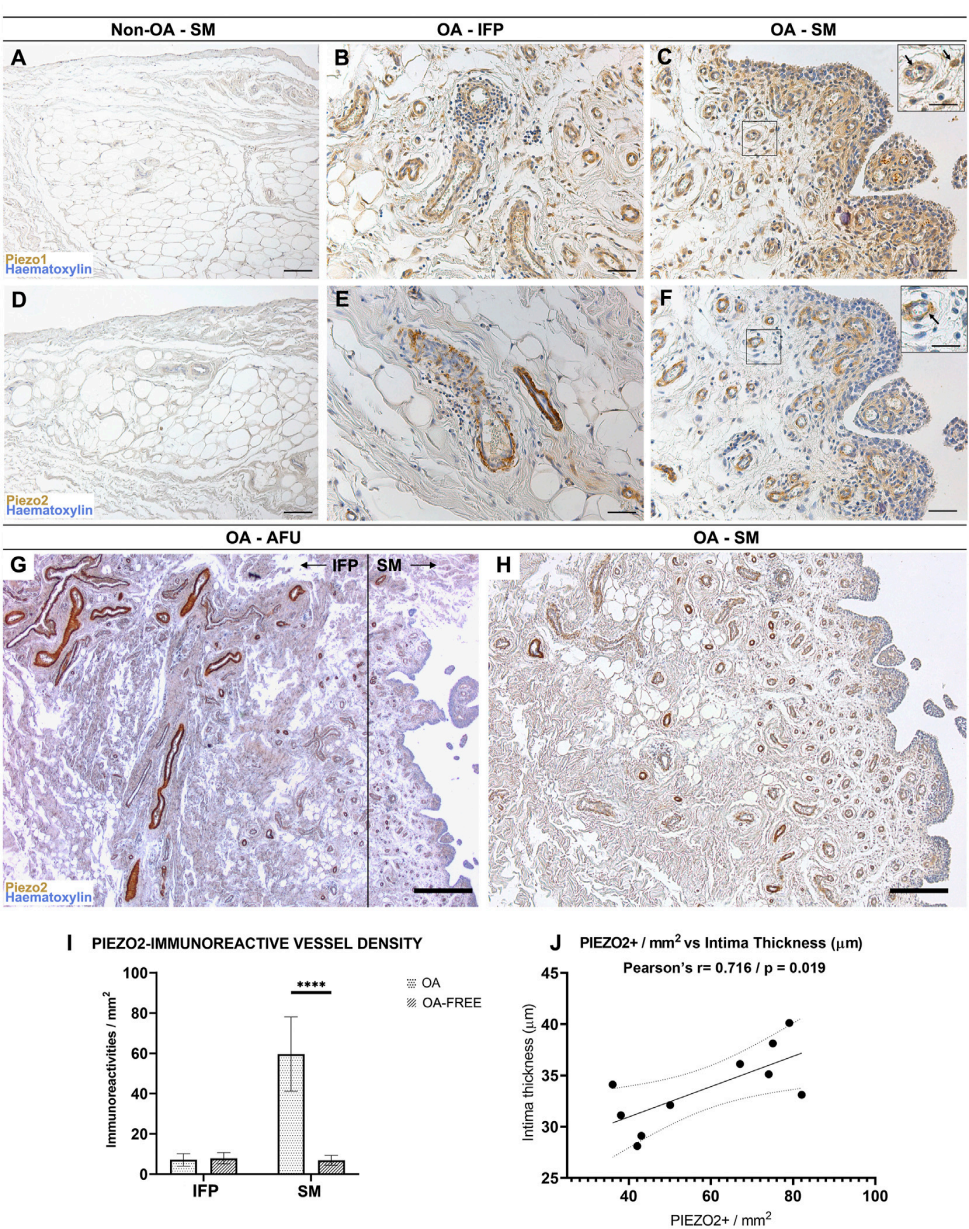
**(A**–**C)** Piezo1 and **(D**–**F)** Piezo2 receptors pattern distribution within the non-OA **(A**,**D)** and diseased **(B**,**C**,**E**,**F)** AFUs by immunohistochemistry. **(G**,**H)** Decreasing IFP-to-synovial membrane gradient of Piezo2 immunoreactivity. Higher immunoreactivity is found in IFP vessels and gradually decreases towards the subintima of the synovia; the dotted line in **(G)** shows the boundary between IFP and SM. **(I)** Evaluation of Piezo2 immunoreactive vessel density in the IFP and SM compartments of the AFUs in OA versus non-OA subjects. Piezo2 vessel density was statistically significantly higher for OA (****p < 0.0001), but not for OA-free subjects. **(J)** A positive correlation (r = 0.716, *p* = 0.019) between intima thickness and Piezo2 immunoreactive vessel density in the subintima was found for OA subjects. Scale bars: 100 µm **(A**,**C**,**D**,**F)**; 50 µm **(B**,**E)**; 400 µm **(G)**; 200 µm **(H)**. Scale bars inserts: 25 µm.

Piezo1/2 mechanoreceptors antibodies showed a peculiar reactivity pattern (brown staining) at the level of the IFP and SM, in the two experimental groups (Figure 3).

##### 3.3.3.1 Piezo1

Non-OA IFP-SM AFU group: considering vessels, a mild immunoreactivity was evidenced at the smooth muscle cells within the IFP, while no positive reaction was detected in the intimal and subintimal layers of the SM (Figure 3A).

OA IFP-SM AFU group: vessels in the IFP and SM compartments (subintimal layer) presented a Piezo1 moderate reactivity, also showing frequent lympho-monocytic infiltrations (Figures 3B,C). Furthermore, also sparse cellular profiles in the OA subintima were mildly reactive; these were morphologically consistent with fibroblast-like type B synoviocytes (Figure 3C).

##### 3.3.3.2 Piezo2

Non-OA IFP-SM AFUs group: as for vessels, mild Piezo2 immunoreactivity was observed in the IFP but not in the SM compartment (Figure 3D). Conversely, in the OA IFP-SM AFU samples (Figures 3E,F), a decreasing immunoreactivity gradient from the IFP towards the SM was noticed, with subintimal vessels being moderately reactive (Figures 3G,H). Unlike Piezo1, no cellular profiles were Piezo2 immunopositive (Figure 3F).

Piezo2 immunoreactivity was also quantified and normalized per mm^2^ (Figure 3I): statistically significant differences (*p* < 0.0001) were detected between OA and non-OA IFP-SM AFUs in the synovial compartment (but not in the IFP) as well as comparing the IFP and the SM of the OA IFP-SM AFUs. Regarding distribution, Piezo2 immunoreactive vessels were more densely represented in the subintima of the SM than in the IFP, but only in the OA group. A strong correlation between Piezo2+ vessel density in the subintima and intima thickness was also detected for OA subjects (r = 0.71, *p* = 0.019; Figure 3J).

#### 3.3.2 CD68 Broad Distribution in the Anatomo-Functional Units

CD68 is a marker employed to distinguish monocyte lineage derivates among leucocytes; furtherly, it is also associated to a certain cellular phagocytic activity.

**FIGURE 4:**
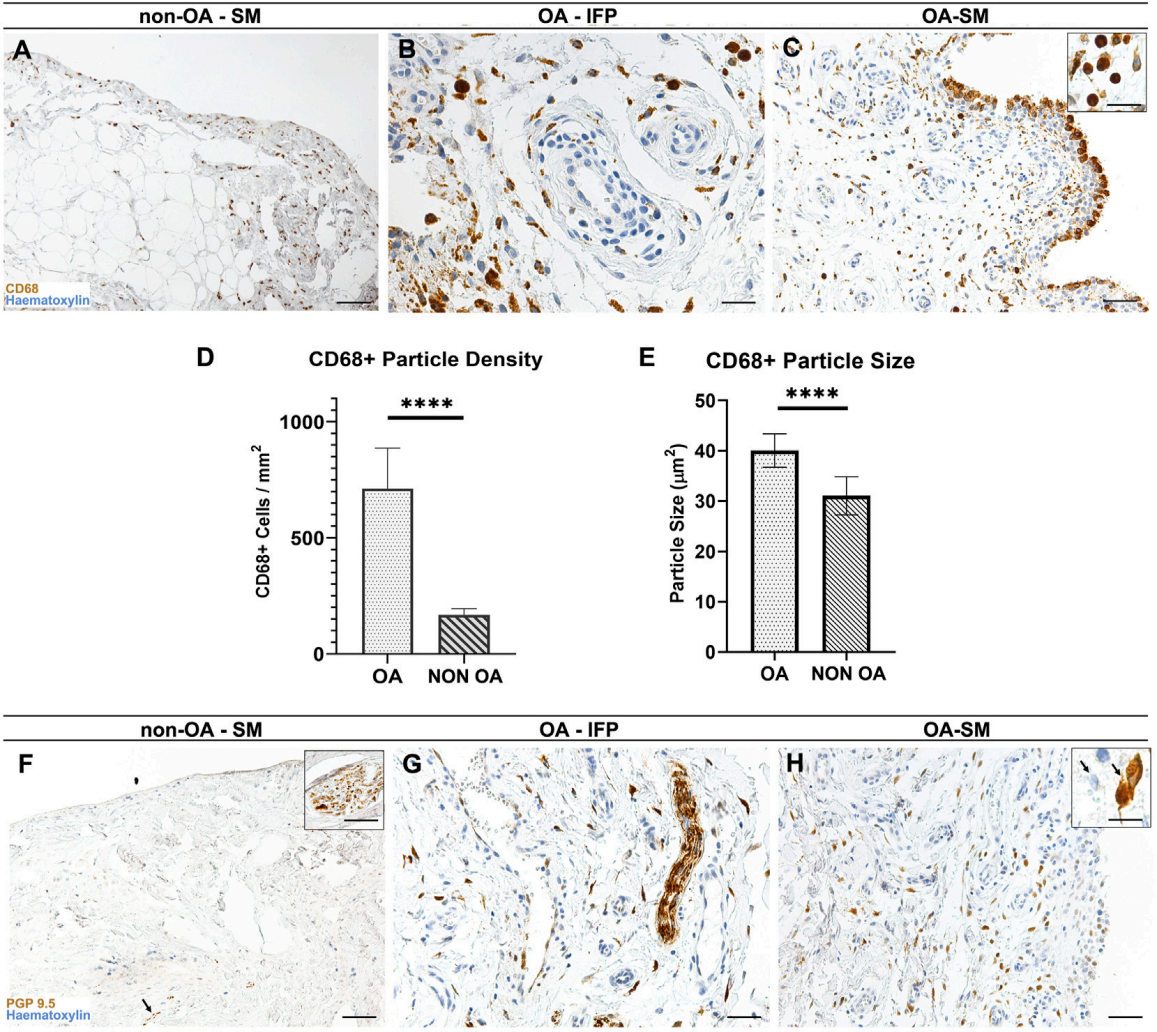
**(A**–**C)** CD68^+^ pattern distribution within the non-OA **(A)** and diseased **(B**,**C)** AFUs by immunohistochemistry. **(D**, **E)** CD68^+^ elements characteristics by morphometric analysis: Welsch-corrected *t*-test of CD68^+^ particle size **(D)** and density **(E)** reveals statistically significant differences between OA and OA-free subjects (****p < 0.0001). **(F**–**H)** PGP9.5 immunoreactivity in the healthy **(F)** and diseased **(G**, **H)** AFUs. Scale bars: 100 µm **(A**,**C**,**F**,**H)**; 50 µm **(B**,**G)**. Scale bars inserts: 25 µm.

Both groups displayed CD68^+^ elements in the IFP tissue and in the intimal and subintimal layers of the SM (Figures 4A–C). In the OA group, immunoreactivity in the subintimal layer was ascribable to monocyte-derived macrophages; however, numerous cells displaying distinct cytoplasmatic CD68 reactivity and morphologically compatible with type A synoviocytes were detected in the most superficial layer of the intima (Figure 4C); this feature was not observed in the non-OA SM.

Lympho-monocytic perivascular cuffing in OA group was only partially reactive for CD68, suggesting for a more prominent of involvement of lymphocytes in the perivascular compartment (Figure 4B).

According to the morphometric study, the SM compartment within the OA-SM-IFP AFUs showed higher CD68^+^ immunoreactive cells than in the non-OA SM-IFP AFUs (711.7 ± 175.1 interval ratio (IR)-cells/mm^2^ versus 167.4 ± 27.91 IR-cells/mm^2^, *p* < 0.0001), with markedly larger overall cell size (40 ± 3.3 µm^2^ versus 31 ± 3.7 µm^2^, *p* < 0.0001) (Figures 4D,E).

#### 3.3.3 PGP9.5 Immunoreactivity in Nerve Fibers and Osteoarthritis-Group Type B Synoviocytes

In both groups a marked immunoreaction towards PGP9.5 was observed in correspondence of the nerve fibers within the IFP and SM compartments (Figures 4F–H); additionally, numerous intimal and subintimal reactive cellular profiles, morphologically compatible with type B synoviocytes and strongly reactive towards PGP9.5 were detected within the OA IFP-SM AFUs (Figure 4H).

#### 3.3.4 YAP1 Only Localizes in the Vessels of the Osteoarthritis-Anatomo-Functional Units Group

**FIGURE 5:**
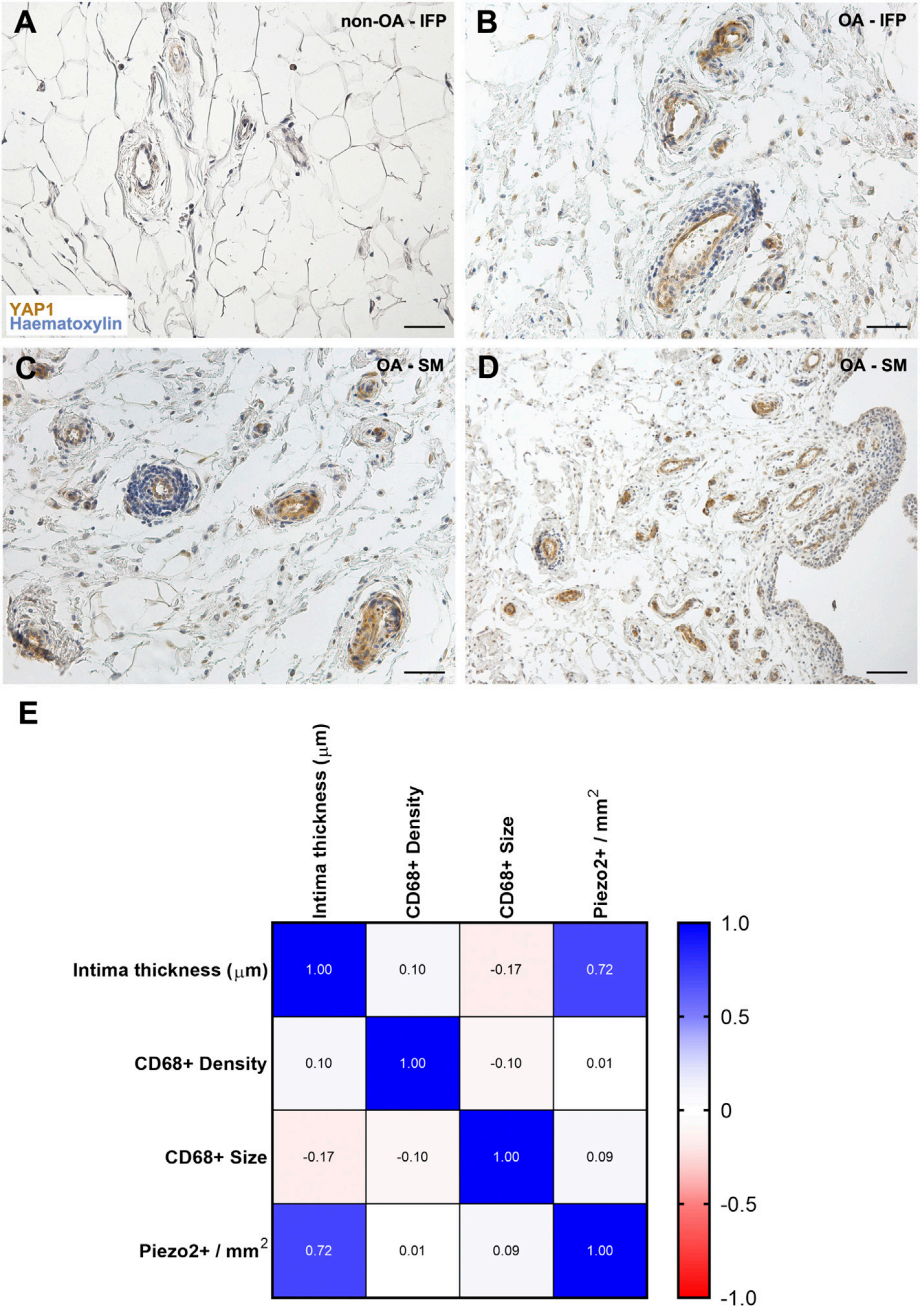
**(A**–**D)** YAP1 pattern distribution within the non-OA **(A)** and diseased **(B**–**D)** AFUs by immunohistochemistry. **(E)** Correlation matrix reveals a positive correlation only between intima thickness and Piezo2+ vessel density, while no relationship was found between the other studied variables. Scale bars: 50 µm **(A**–**C)**; 100 µm **(D)**.

Regarding YAP1 (Figures 5A–D), a different distribution was observed within the cohort, as it was only displayed by the vessels wall within both the IFP and the SM (intima and subintima) in the OA-group. No reactivity was detected in the non-OA group.

### 3.4 Correlations

A correlation matrix between SM intima thickness, Piezo2+ vessels density, CD68^+^ cells size and density were developed (Figure 5E). According to experimental data, only a significant correlation was found between intimal thickness and Piezo2+ vessel density (r = 0.71, *p* = 0.019).

## 4 Discussion

The new paradigm in OA description has profound anatomical roots, being anchored on awareness that the knee structures experience together dynamic active/passive changes in a “dialogue of equals” where the IFP-SM AFU well summarizes this interplay (Macchi et al., 2018). Thus, elucidating the anatomically-based cross-talk within the IFP-SM AFU may help to define a new methodological approach for OA-joint interpretation, and also fulfill the gap in OA comprehension and pain management (Defrate et al., 2019; Hunt et al., 2020), unravelling the existing crosstalk among mechanoreception, inflammation and pain.

Pain is the multifactorial hallmark symptom of OA, a disease typically characterized by imbalance of anabolism and catabolism in the cartilage joint and influenced by the biological and mechanical environment (Poulet, 2016; Belluzzi et al., 2019b). It results from a combination of risks factors, with increasing age and obesity being the most prominent: high BMI is a common condition (Martel-Pelletier et al., 2016; Belluzzi et al., 2017). The OA-patients enrolled in this study had a mean BMI of 35.41 kg/m^2^ (obesity, class II): it is easily inferable that excess weight, together with motor adaptations, caused an increased joint loading and mechanical stress with consequent detrimental effects over the knee (Kulkarni et al., 2016; Xu et al., 2020; Sun et al., 2021). Whereas physiologic loading is fundamental to guarantee healthy joint maintenance, improper mechanical loading (i.e., compression, tension, shear) together with circulating obesity-related inflammatory cytokines are major risks factors for OA, determining irreversible homeostasis disruption and structural/ultrastructural tissue changes (Guilak, 2011; Henak et al., 2013; Fontanella et al., 2018; Wang and He, 2018; Defrate et al., 2019; Belluzzi et al., 2020; Xu et al., 2020; Jeong et al., 2021; Sun et al., 2021) like that highlighted within the OA IFP-SM AFUs group.

In accordance with evidence reported in the literature (Sellam and Berenbaum, 2010; Scanzello and Goldring, 2012; Favero et al., 2017; Fontanella et al., 2018), the histopathological analysis of the OA IFP-SM AFUs confirmed the typical microscopic OA-related features with increased inflammatory cells and vessels. OA-histopathological signs were assessed in both IFP and adjacent SM compartments, suggesting that their anatomical proximity and biochemical interaction might lead to mutual conditioning (Macchi et al., 2018). Regarding the SM, the thickening of both the subintimal and synovial lining cells layer was only observed in OA-SM versus the non-OA samples. Hyperplasticity and fibrosis were reported to exert a significant contributory role in joint pain and stiffness (Vaamonde-Garcia et al., 2019): resident synoviocytes are active in the tissue homeostatic function including excretion of synovial fluid factors, intra-articular debris clearance, control over immunological events but, due to their fibroblast lineage (i.e., fibroblast-like synoviocytes type B), can also be directly involved in OA pathogenesis, triggering excessive ECM deposition or decreased degradation (Steenvoorden et al., 2006; Remst et al., 2015; Maglaviceanu et al., 2021). As for the IFP, typical thickened septa may derive from the protective activity of OA-IFP stem cells towards mechanical overloading: mesenchymal stem cells can sense both mechanical stress and soluble stimuli and translate such information in adaptive responses (Stocco et al., 2019) in turn affecting the mechanical behaviour of the tissue. Possibly, IFP fibrosis is responsible of tissue modifications, altering the normal forces distribution within the joint during movement with consequent disease worsening and pain (Favero et al., 2017; Belluzzi et al., 2020; Fontanella et al., 2022). Considering the control group, in accordance with the inclusion/exclusion criteria, no history of symptomatic OA, knee comorbidities, rheumatic/inflammatory disorders, tumors, signs of cartilage degradation or presence of osteophytes on dissection was reported for the donors. Histological evaluations furtherly corroborated this evidence later. In addition to mechanical overloading, articular inflammatory environment (also descending from a chronic low-grade inflammatory condition) has a leading role in OA pathogenesis and progression too (Griffin and Scanzello, 2019; Wu et al., 2020; Zhang et al., 2020). Macrophages are deeply involved in many of the following inflammation-related events: leukocyte/lymphocyte recruitment, fibroblast proliferation, protease secretion and angiogenesis stimulation, which in turn promotes infiltration at the injury site of cytokines and immune cells (Kennedy et al., 2011; Jeong et al., 2021). To this purpose, presence and distribution of CD68^+^ cells were specifically considered within the study cohort and experimental evidence, supported by the literature, suggested their possible different origin. CD68 immunopositivity was moderate at the vascular cuffing of OA-AFUs (in both IFP and SM), while predominant throughout the sub-lining layer and within the lining layer at SM, thus distinguishing as a specific OA-AFU feature. In accordance with Mucke et al., we hypothesized that this localization may derive from an increase in extravasation of precursor cells from the blood, with more rapid homing towards the lining layer (Mucke et al., 2016). Moreover, the increased vessels number detected within the OA IFP-SM AFUs and typically occurring in an inflamed environment supports this hypothesis. However, synovial CD68^+^ macrophages are also typical SM resident cells that are quiescent in the healthy joint and get activated as a consequence of joint inflammation, in turn contributing to the production of inflammatory mediators and tissue degrading proteinases; these events drive tissue histological changes determining cartilage/subchondral bone destruction and consequent higher self-reported pain (Sellam and Berenbaum, 2010; Kennedy et al., 2011; Chen et al., 2020; Zhang et al., 2020). Also type A synoviocytes are CD68^+^ elements and immunopositivity for CD68 corroborates their phagocytic activity within the SM compartment (Ando et al., 2010; Li et al., 2019).

The link between inflammation and vascularization was confirmed within the study cohort; as stated above, the OA IFP-SM AFUs displayed a significantly higher number of vessels compared to the non -OA group, in accordance with our previous studies (Favero et al., 2017; Belluzzi et al., 2020). Together with hyperplasticity, fibrosis and CD68^+^ cells recruitment/activation, also the presence of blood vessels can directly represent a source of pain, amenable to vascular endothelium (Cook et al., 2005; Mapp and Walsh, 2012). Endothelial cells produce endothelins (ETs), contain endothelin receptors (ET_A_ and ET_B_) (also located on nociceptors) and release ATP in response to mechanical stimulation, which in turn can act on P2X_2/3_ receptors (Joseph et al., 2013; Joseph et al., 2014). Hence ET-1 mediates 1) primary hyperalgesia, by its action on the peripheral terminal of nociceptors; 2) stimulus induced-enhancement of endothelin hyperalgesia, by its action on vascular endothelial cells, sensitizing them for mechanical stimulus-induced release of pronociceptive mediators (Joseph et al., 2013; Joseph et al., 2014). Within this scenario, to furtherly elucidate the mechanism correlating increased vascularization with mechanotransduction and pain, the presence of Piezo1/2 ion channels was assessed in the IFP-SM AFU.

Piezo channels are mechanically activated proteins mainly expressed in highly stimulated tissues (e.g., lung, colon, bladder, kidney, blood vessels, cartilage and ganglia), sensing compression load and tensile load. Likely, they have a synergistic effect in conveying mechanical signals, with possible involvement in harmful mechanical stimuli transmission (Xu et al., 2020). Typically, their expression is potentiated by inflammation (Lee et al., 2014; Borbiro and Rohacs, 2017; Lee et al., 2017; Lee et al., 2021). Considering the mechanical role of the IFP and its contributory role to inflammation, Piezo receptors’ possible presence in this tissue as well as in the SM (due to anatomical and functional contiguity/relation, being part of the AFU) has been considered in this study, while receptors’ presence assessment is a key element for future functional studies. Piezo1 was moderately represented in both the OA-IFP vessels (slightly in non-OA AFUs) and OA-SM vessels (not detected in non-OA AFUs); it is implied in vascular remodeling, it is sensitive to flow-induced shear stress with a role in endothelial cells alignment (Ranade et al., 2014; Lai et al., 2021). Its presence suggests AFU plasticity. A stiffer mechanical microenvironment (like the fibrotic one in the OA) triggers its overexpression; however, it can also be directly involved in scar tissue formation (He et al., 2021). Together with Piezo1 receptor, also YAP1 expression suggests vascular remodeling (Yamashiro et al., 2020; Osman et al., 2021; Wang X. et al., 2022). Specifically, YAP1 signaling, modulated by mechanical stimuli intensity, is critical in regulating endothelial/vascular smooth muscle cells behaviour (i.e., survival, proliferation, migration, apoptosis) (He et al., 2018; Wang X. et al., 2022). Importantly, only OA-IFP-SM AFU vessels displayed moderate YAP1 expression, suggesting that also vascular remodeling is an OA feature. Further studies are needed to elucidate the role of vascular remodeling in OA.

Regarding Piezo2, its distribution resembles that of Piezo1, being moderate in the OA-SM vessels (not detected in non-OA AFUs), whereas a strong immunoreactivity was highlighted in the IFP-compartment (slight in non-OA AFUs). Piezo2 mediates mechanotransduction in the somatosensation of touch, proprioception, and pain (Guo et al., 2021). Intriguingly, Ferrari et al. (2015) demonstrated that Piezo2 is a mechano-transducer in the endothelial cell where it contributes to stimulus-dependent hyperalgesia in endothelium-related pain induced by innocuous stimuli (Ferrari et al., 2015). Considering the important differences in Piezo1/2 expression comparing OA and non-OA AFUs, and their marked expression in OA subjects, our data support the role of vascular mechanisms in OA pain, with Piezo2 playing a potential role in mediating enhancement of pain sensitization. Furthermore, the OA IFP-to-SM decreasing gradient in Piezo2 ion channel expression hints towards a prominent role of the IFP compartment in OA. Thus, this peculiar adipose tissue is actively involved not only in the transduction of mechanical stimuli (Fontanella et al., 2018; Fontanella et al., 2022) but also in mediating chronic pain. Therefore, modulating Piezo receptors expression/activity may help in reducing the physiological painful response deriving from their stimulation, paving the road for new treatment strategies and interventions.

In addition to central nociceptive pathways, the crosstalk between the immune system and the peripheral nociceptive neurons contributes to sustain OA pain too (Conaghan et al., 2019; Miller et al., 2020; Wang D. et al., 2022). Blood vessel and nerve growth are bounded by common pathways that involve the release of both proangiogenic factors and neuropeptides. Proangiogenic factors might also have a role in nerve growth stimulation, and vascular cells-derived molecules could both stimulate and guide nerve growth (Mapp and Walsh, 2012). To enhance consciousness on the sensory innervation role in OA joint pain, the anatomical distribution of free-nerve endings was assessed within the IFP-SM AFUs through pan-neuronal marker PGP9.5 immunostaining. While in healthy tissues its presence was rare, a highest density was detected in both the SM and IFP compartments of the OA-AFUs. Interestingly, together with nerve fibers, also cellular elements resembling type B synoviocytes were positively immunoreactive to PGP9.5. According to our knowledge, this is the first evidence of PGP9.5 positive synoviocytes in human specimens. A similar pattern was only evidenced by Kitamura et al. (1999) in animal-derived samples (Kitamura et al., 1999). Additionally, PGP9.5 positive cellular elements increased in OA-SM-IFP AFUs, thus suggesting this protein as a potential biomarker for arthritic disorders (Kitamura et al., 2001).

**FIGURE 6:**
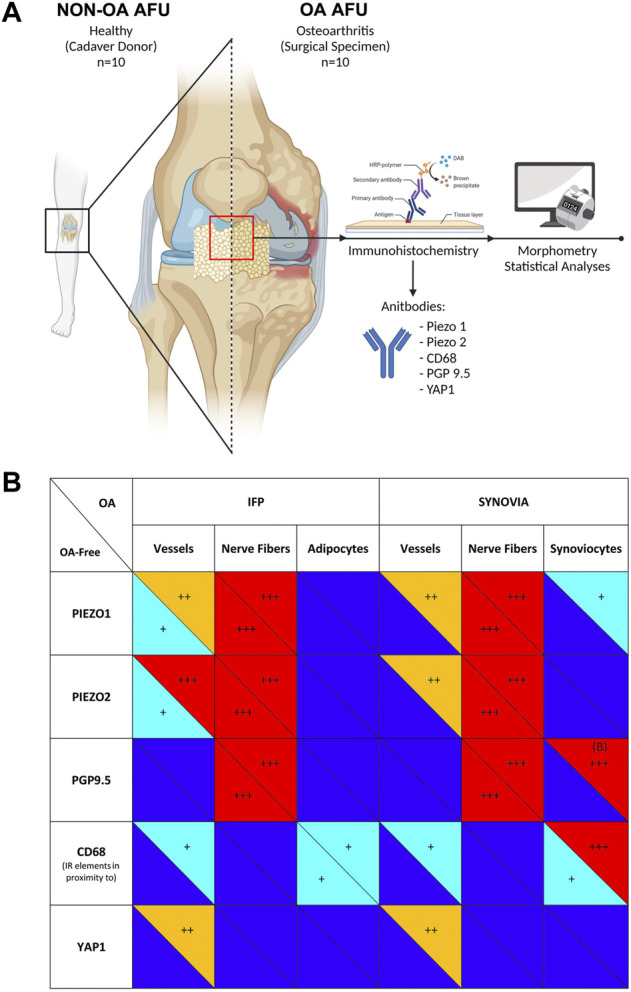
**(A)** Pipeline of the study. The Anatomo Functional Units (AFUs) specimens were sampled from Donors of the Body Donation Program of the University of Padova (non-OA-AFU, *n* = 10) or patients with Osteoarthritis (OA AFU) during surgical procedures at the Orthopedics and Orthopedic Oncology, University-Hospital of Padova (*n* = 10). Specimens were routinely processed and underwent immunoperoxidase staining. Digitally assisted morphometrical analyses were employed to quantify immunoreactivity and immunoreactive structures within the infrapatellar fat pad (IFP) and synovial membrane (SM) compartments. **(B)** Immunoperoxidase staining intensity for the different antibodies was graded as follows: +++ marked (red); ++ moderate (orange); + mild (light blue); no staining (dark blue). Panel A was created with BioRender.com.

To date, due to therapies ineffectiveness in reducing OA progression, the gold standard for knee OA pain/disability resolutive management remains TKA (Zeni et al., 2010). However, it is still under debate if IFP removal during TKA affects patient outcome (Yao et al., 2021). Therefore, only broad consciousness on anatomy, OA pain-related mechanisms, dynamic tissues modifications and joint tissues crosstalk will help researchers and clinicians in a better patient targeting (O’neill and Felson, 2018). In this study, the IFP and the SM compartments were considered as part of the AFU and their OA-related behaviour as well as mutual conditioning was analyzed. Specifically, Piezo1/2 mechanosensors, CD68 inflammatory marker, PGP9.5 neuronal marker and YAP1 angiogenesis-related marker expression were considered in diseased versus healthy tissues. According to our evidence (Figure 6), together with possible identification in PGP9.5 as a biomarker for OA disease, we also demonstrated Piezo expression within the IFP-SM AFU for the first time. Assessing Piezo 1/2 presence is a prerequisite for subsequent studies including dedicated mechanical and functional evaluations that will be helpful to better unravel Piezos’ role *in vivo*; furtherly, description of Piezo receptors pattern in patients with a matched BMI might be of particular interest too, allowing to effectively discriminate loading contribution to histopathological joint tissues features.

Possibly, as Piezo2 is involved in stimulus-dependent hyperalgesia, controlling its expression (and eventual implication with cartilage specific features in OA), may contribute to future development of therapies able to guarantee an effective pain management, with consequent significant amelioration in patients’ life quality.

## Data Availability

The raw data supporting the conclusions of this article will be made available by the authors, without undue reservation.
